# Impact of 24-epibrassinolide, spermine, and silicon on plant growth, antioxidant defense systems, and osmolyte accumulation of maize under water stress

**DOI:** 10.1038/s41598-022-18229-1

**Published:** 2022-08-27

**Authors:** Azizolah Ghasemi, Salim Farzaneh, Sajjad Moharramnejad, Raouf Seyed Sharifi, Ahmed Fathy Youesf, Arkadiusz Telesinski, Hazem M. Kalaji, Jacek Mojski

**Affiliations:** 1 Department of Genetic and Plant Production, Faculty of Agriculture and Natural Resources, University of Mohaghegh Ardabili, Ardabil, Iran; 2 Crop and Horticultural Science Research Department, Ardabil Agricultural and Natural Resources Research and Education Center, AREEO, Moghan, Iran; 3 Department of Horticulture, College of Agriculture, University of Al-Azhar (Branch Assiut), Assiut, 71524 Egypt; 4 Department of Bioengineering, West Pomeranian University of Technology in Szczecin, 17 Słowackiego Street, 71-434 Szczecin, Poland; 5 Department of Plant Physiology, Institute of Biology, Warsaw University of Life Sciences SGGW, 02-776 Warsaw, Poland; 6 Institute of Technology and Life Sciences, National Research Institute, Falenty, Al. Hrabska 3, 05-090 Raszyn, Poland; 7 Twój Swiat Jacek Mojski, ulica Okrzei 39, 21-400 Lukow, Poland; 8 Fundacja Zielona Infrastruktura, ulica Wiatraki 3E, 21-400 Lukow, Poland

**Keywords:** Plant sciences, Environmental sciences, Chemistry

## Abstract

The effect of triad application of the phytohormone 24-epibrassinolide (EBL), the polyamine spermine (Spm), and the element silicon (Si) has not yet been considered on plant growth and behavior in water-stressed conditions. We aimed to evaluate the impact of single/dual/triad application of 24-epibrassinolide (EBL), spermine (Spm), and silicon (Si) on the growth, photosynthetic metabolites, and antioxidant enzymes in the maize plant exposed to water stress. This study was conducted as a potential drought resistance system and plants' maintenance against oxidative damage. In this regard, one maize hybrid (Paya) was grown under well-watered and water-deficit conditions (interrupted irrigation at the flowering and the filling seed stages) with and without foliar spraying of EBL, Spm, and/or Si. Drought conditions remarkably reduced growth, productivity, water-related content (RWC), and chlorophyll content. However, the dual and triad applications of EBL (0.1 mg L^−1^), Spm (25 mg L^−1^), and Si (7 mg L^−1^) significantly improved the above parameters. Water stress considerably augmented the levels of H_2_O_2_ and MDA. Their content in stress-subjected plants was significantly reduced by triad application. In water-stressed circumstances and after foliar treatments, the activities of superoxide dismutase, catalase, and peroxidase as well as the amounts of total soluble proteins, phenolic compounds, proline, and glycine betaine all improved. Overall, triad application increased the plant's drought resistance and diminished ROS accumulation by raising the scavenging via the enhanced activity of the antioxidant enzymes.

## Introduction

Drought is the main cause of environmental stress conditions, which negatively affects plants' growth and productivity. It also influences a broad spectrum of physiological and molecular aspects of plant^1^. Plants have developed several approaches, such as producing compatible solutes to evade the damage caused by water stress^2^. Accumulation of organic solutes reduces oxidative damage under drought conditions, safeguards subcellular structures, and maintains enzyme activity^3,4^. Additionally, reactive oxygen species (ROS) (e.g., superoxide radical, hydrogen peroxide, and hydroxyl radical) are excessively generated under stress conditions. ROS production induces lipid peroxidation, membrane disruption, DNA modifications, and thus metabolic and structural dysfunctions^5,6^. The plant develops an antioxidant defense system as an organized response to face ROS and to preserve redox homeostasis i.e. they produce antioxidant molecules/enzymes of catalase, peroxidase, and superoxide dismutase. Because they reduce oxidative damage, these antioxidant enzymes are very effective in the presence of abiotic stress^7,8^.

Modern agriculture faces the enormous challenge of enhancing plant drought tolerance through polyamines. Plant polyamines (PAs) critically contribute to the plant to cope with various environmental stresses^1,4^. Growing evidence of endogenous polyamines in plants cultivated in water-stressed situations points to their potential contribution to that stress. Moreover, in the plants with more amino groups such as spermine (Spm) and spermidine (Spd) (rather than putrescine), ROS were highly scavenged^9,10^. Additionally, depending on the plant's genotype, different polyamines have different profiles and concentrations. A drought-tolerant cultivar has much higher levels of free Spm and Spd, whereas a drought-sensitive cultivar has higher levels of free putrescine^1,10^. Alterations in the synthesis and catabolism of PAs have also been detected in stress conditions^11^. Correspondingly, a reduction in protein content and a raise in protein carbonyl levels have been observed in the plants affected by diverse stresses^12^.

Plant hormones known as brassinosteroids (BRs) control a variety of processes in plants, such as cell division, photosynthesis, reproductive development, enzyme activation, protein/gene synthesis, and expression. BRs have a critical role in the plant's tolerance to the stress conditions^13^. The basic processes behind BR-mediated growth/development and stress response in plants are not fully understood yet, despite the substantial attempts to employ BRs as plant growth regulators in agriculture. Under water stress conditions, application of 24-epibrassinolide (EBL) is shown to modify the activity of antioxidant enzymes, accumulation of osmoprotectants, level of antioxidant molecules and H_2_O_2_, lipid peroxidation, and membrane stability in plants^14,15^.

The highly abundant element, Si, is found in combined (not free) form with other features, usually as oxides, and its absorption by the plant is as Si (OH)_4_. Si has not commonly been introduced as an essential element in higher plants. This is partially because the data to far does not adequately establish Si as a crucial component or metabolite in plants^16^. However, the importance of Si has been recently delineated in enhancing the plant response to biotic as well as abiotic stresses^16,17^.

Maize (*Zea mays* L.) is a food crop susceptible to suboptimal conditions, even moderate levels of drought. Growth and productivity in the corn are adversely affected by drought, during which osmotic changes occur due to the limited availability of water.

Spermine (Spm) as the effective polyamine in enhancing the plant tolerance to drought efficiently neutralizes and stabilizes the membrane in plants^9^. Further, 24-epibrassinolide (EBL), greatly contributes to improving the plant response to different stresses, e.g., pathogen infection, drought, oxidative, thermal, and heavy metal stress. Although oxidative stress and BR accumulation are significantly associated, little is known about the physiological processes behind BR changes^18^. Si has been proven to improve the tolerance of maize to drought stress^19^. Exogenous application of EBL^15^, Spm^1^, and Si^16^ has a significant role in plant tolerance to abiotic stress. They play their role in water stress by activating the antioxidant enzymes, elevating antioxidant levels, and reducing MDA, O_2_^**.**−^ as well as H_2_O_2_. Although much is known about the role of EBL, Spm, and Si in the development and responses regulated in plants exposed to stress are not completely characterized. We investigated the triple effect of EBL, Spm, and Si on plant tolerance against drought stress for the first time. The current research was conducted to evaluate the single/dual/triad foliar application of EBL, Spm, or Si on neutralizing adverse effects in a plant by drought stress. In this respect, the executive strategies of the defensive responses—pigment content, osmolyte accumulation, and antioxidant defence system scavenging capacity—were tracked. For this purpose, maize cultivar (Paya, FAO-700) was treated with or without Si, Spm, and/or EBL under both conditions of full irrigation and drought stress. Afterward, several physiological parameters were measured, including the changes in the activity of antioxidant enzymes (CAT, POX, and SOD), photosynthetic pigment, osmoprotectants of proline and glycine betaine, in addition to MDA and H_2_O_2_. The present investigation aimed to uncover the relationship between measured features and the level of plant tolerance regarding its growth and productivity. The ultimate aim of this research is to get a deep insight into the mechanisms underlying drought tolerance in the exposed plants by defining the impact of EBL, Spm, and/or Si on specific physiological characters.

## Results

**Figure 1:**
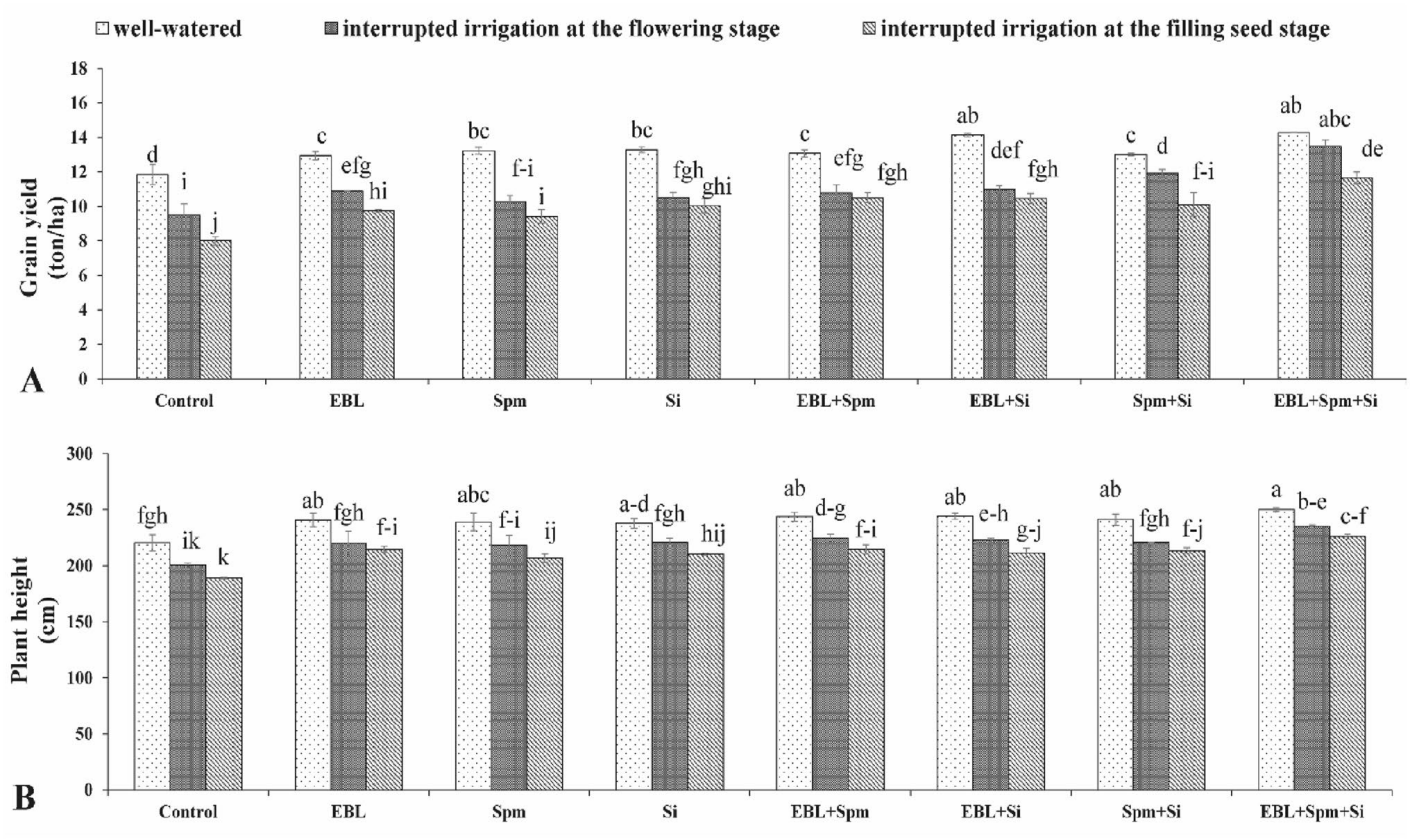
Changes in the grain yield (**A**) and plant height (**B**) were exposed for various foliar applications [00.00 (double distilled water), 0.1 mg L^−1^ EBL, 25 mg L^−1^ Spm, 7 mg L^−1^ Si, 0.1 mg L^−1^ EBL + 25 mg L^−1^ Spm, 0.1 mg L^−1^ EBL + 7 mg L^−1^ Si, 25 mg L^−1^ Spm + 7 mg L^−1^ Si, and 0.1 mg L^−1^ EBL + 25 mg L^−1^ Spm + 7 mg L^−1^ Si] under different water deficit stress (well-watered condition and water-stressed conditions as interrupted irrigation at the flowering and the filling seed stages). Value shown in each column is the mean of three replicates ± standard error. Different letters indicate significant differences among treatments according to LSD test when p ≤ 0.05. Where: *EBL* 24-epibrassinolide, *Spm* spermine, and *Si* silicon.

Grain yield and plant height were negatively affected (*p *< 0.05) by water-stressed compared to non-stressed conditions (Fig. 1). However, treatment by 24-epibrassinolide (EBL), spermine (Spm), and/or silicon (Si) improved plant growth and production in water-deficit and well-watered conditions. With the triad application of EBL, Spm, and Si, stressed plants were able to reach their optimum plant height and grain output. This demonstrates the positive role of the mentioned treatment in tolerance to drought. When EBL, Spm, and Si were applied as a trio, the growth was significantly enhanced under water-stressed circumstances as opposed to under non-stressed conditions. Under triad application of EBL, Spm, and Si, the plant height in hybrid Paya was increased by 13.3%, 17.0%, and 20.0% in comparison to untreated plants under non-stressed and stressed conditions of interrupted irrigation at the flowering and the filling seed stages, respectively. The corresponding values of the grain yield were also improved by 20.4%, 41.9%, and 45.5%.

**Figure 2:**
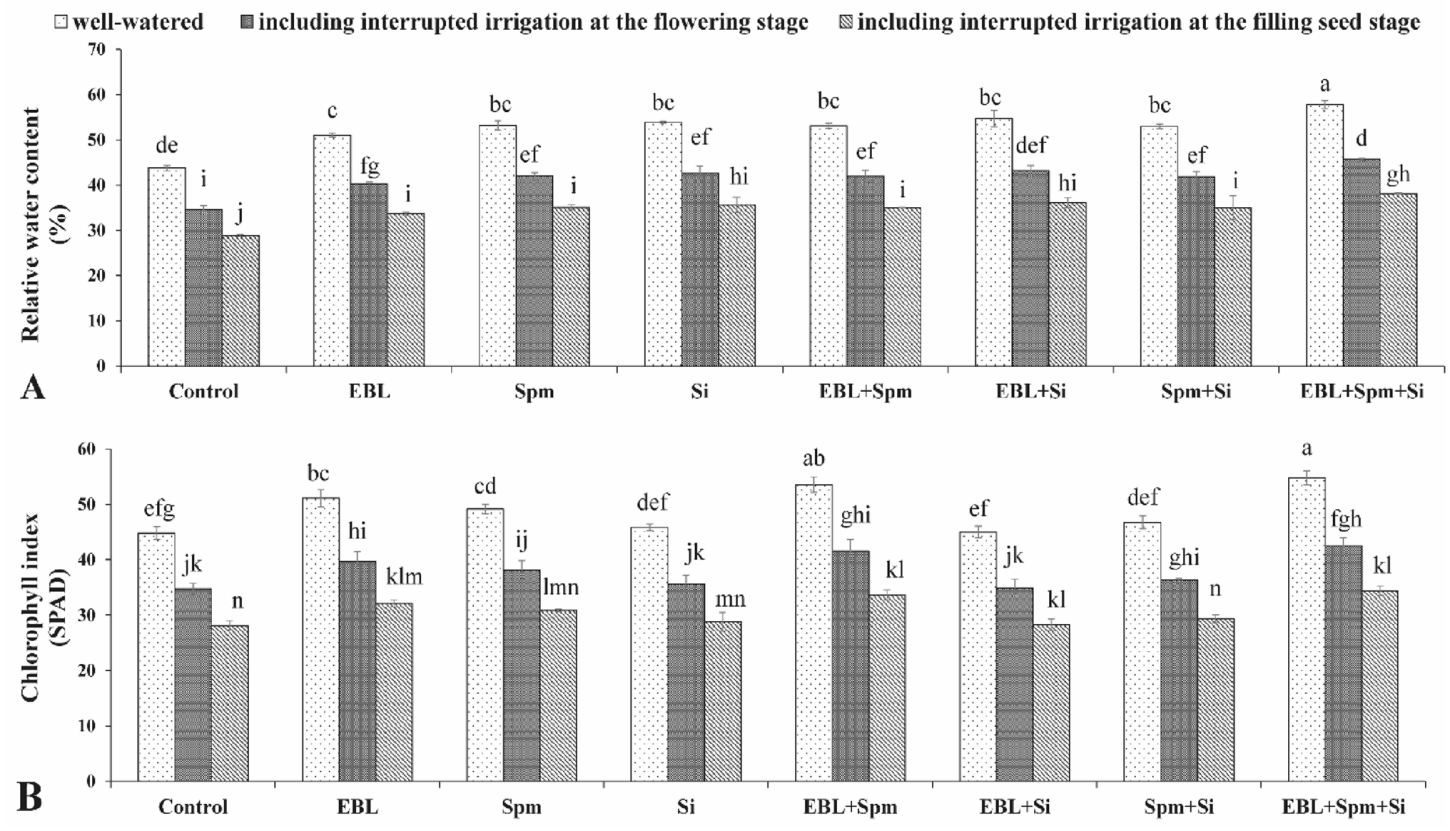
Changes in the relative water content (RWC) (**A**) and chlorophyll index (**B**) were exposed for various foliar applications [00.00 (double distilled water), 0.1 mg L^−1^ EBL, 25 mg L^−1^ Spm, 7 mg L^−1^ Si, 0.1 mg L^−1^ EBL + 25 mg L^−1^ Spm, 0.1 mg L^−1^ EBL + 7 mg L^−1^ Si, 25 mg L^−1^ Spm + 7 mg L^−1^ Si, and 0.1 mg L^−1^ EBL + 25 mg L^−1^ Spm + 7 mg L^−1^ Si] under different water deficit stress (well-watered condition and water-stressed conditions as interrupted irrigation at the flowering and the filling seed stages). Value shown in each column is the mean of three replicates ± standard error. Different letters indicate significant differences among treatments according to LSD test when p ≤ 0.05. Where: *EBL* 24-epibrassinolide, *Spm* spermine, and *Si* silicon.

The water stress significantly decreased (*p* < 0.05) the plant RWC compared to non-stress conditions. At the flowering and filling seed stages, this value in response to drought stress was 21.6% and 34.6% under interrupted watering, respectively (Fig. 2). Further, compared to non-stress condition, water-stress significantly decreased (*p* < 0.05) chlorophyll index. It was measured as 22.4% and 37.6% under drought stress of interrupted irrigation at the flowering and the filling seed stages, respectively (Fig. 2). Nevertheless, the dual/triad application diminished (*p* < 0.05) drought damage and improved RWC as well as chlorophyll index. The most efficient treatment, which elevated RWC and chlorophyll index in the maize leaves, was EBL (0.1 mg L^−1^) + Spm (25 mg L^−1^) + Si (7 mg L^−1^) (Fig. 2).

**Figure 3:**
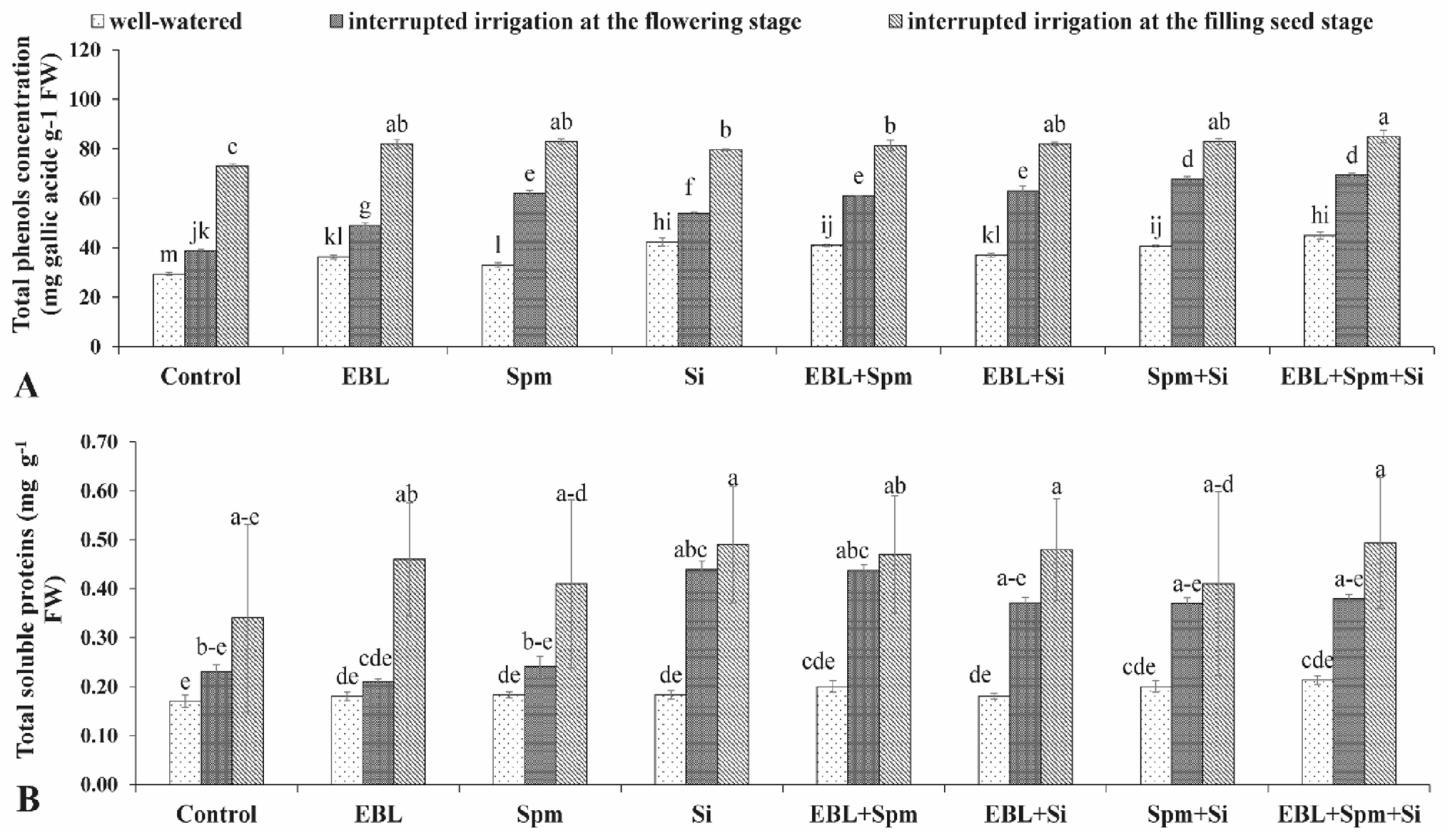
Changes in the total phenols concentration (**A**) and total soluble proteins (**B**) were exposed for various foliar applications [00.00 (double distilled water), 0.1 mg L^−1^ EBL, 25 mg L^−1^ Spm, 7 mg L^−1^ Si, 0.1 mg L^−1^ EBL + 25 mg L^−1^ Spm, 0.1 mg L^−1^ EBL + 7 mg L^−1^ Si, 25 mg L^−1^ Spm + 7 mg L^−1^ Si, and 0.1 mg L^−1^ EBL + 25 mg L^−1^ Spm + 7 mg L^−1^ Si] under different water deficit stress (well-watered condition and water-stressed conditions as interrupted irrigation at the flowering and the filling seed stages). Value shown in each column is the mean of three replicates ± standard error. Different letters indicate significant differences among treatments according to LSD test when p ≤ 0.05. Where: *EBL* 24-epibrassinolide, *Spm* spermine, and *Si* silicon.

Water stress increased the content of phenolic compounds in the plant. This increase was more noticeable in the plants grown under stress and sprayed with EBL, Spm, and/or Si (Fig. 3). The treated plants with EBL (0.1 mg L^−1^), Spm (25 mg L^−1^), and Si (7 mg L^−1^), ideally showed improved phenolic compounds in their leaves by 32.8%, 45.1%, and 50.1% under well-watered and interrupted irrigated condition at the flowering and the filling seed stages compared to untreated plants, respectively. Under water stress, total soluble protein increased, and under drought stress, it significantly improved in plants treated with EBL, Spm, and/or Si (Fig. 3). At the flowering and filling seed stages, triad application—the ideal treatment—significantly enhanced total soluble proteins by 15.4%, 45.64%, and 54.34% in comparison to untreated plants, respectively, when irrigation was discontinued.

Electrophoretic analyses indicated that one, two, and three isoforms were present for CAT, POX, and SOD respectively (Fig. S1). Comparing water stressed to well-watered circumstances, anti-oxidative enzyme activity was altered; drought stress increased CAT, POX, and SOD activities in the hybrid maize (Table 1). Even more increases in their activities occurred in the treatments with EBL, Spm, and/or Si. Triad application ideally elevated the activity of SOD by 36.6% and 55.4%, POX by 56.9% and 62.4%, and CAT by 61.7% and 70.3% under interrupted irrigation at the flowering and the filling seed stages than untreated plants, respectively.

**Table 1:** Changes in the superoxide dismutase (SOD), peroxidase (POX), and catalase (CAT) isoform activities were exposed for various foliar applications [00.00 (double distilled water), 0.1 mg L^−1^ EBL, 25 mg L^−1^ Spm, 7 mg L^−1^ Si, 0.1 mg L^−1^ EBL + 25 mg L^−1^ Spm, 0.1 mg L^−1^ EBL + 7 mg L^−1^ Si, 25 mg L^−1^ Spm + 7 mg L^−1^ Si, and 0.1 mg L^−1^ EBL + 25 mg L^−1^ Spm + 7 mg L^−1^ Si] under different water deficit stress (well-watered condition and water-stressed conditions as interrupted irrigation at the flowering and the filling seed stages).

Water stress	Foliar application	Densitometric activity					
		SOD_1_	SOD_2_	SOD_3_	POX_1_	POX_2_	CAT
Well-watered	Control	830.40 l	880.67 k	1085.77 k	175.73 l	206.43 k	280.63 h
	EBL	866.07 kl	1003.87 ij	1132.50 jk	214.07 k	237.33 j	335.97 gh
	Spm	983.20 ij	964.40 jk	1094.80 k	202.00 k	245.83 ij	345.97 gh
	Si	995.90 ij	1098.40 ghi	1225.83 ij	236.10 j	298.63 fgh	353.97 gh
	EBL + Spm	954.90 jk	1005.57 ij	1248.77 hi	246.03 ij	280.97 h	335.97 gh
	EBL + Si	1128.67 efg	1012.63 ij	1276.33 ghi	258.97ghi	258.10 i	322.60 gh
	Spm + Si	1005.43 hij	1145.17 fgh	1302.33 ghi	277.23 efg	282.70 h	386.47 fg
	EBL + Spm + Si	1068.20 ghi	1154.33 fgh	1362.00 efg	280.00 efg	355.90 b	386.47 fg
Interrupted irrigation at the flowering stage	Control	979.47 ij	1038.67 hij	1280.50 ghi	207.63 k	243.83 ij	331.27 gh
	EBL	1094.93 fgh	1183.90 efg	1335.60 f–i	242.37 ij	290.33 gh	387.03 fg
	Spm	1021.43 hij	1185.97 efg	1291.17 ghi	252.83 hij	284.97 gh	396.53 fg
	Si	1145.93 d–g	1137.43 fgh	1445.60 def	295.60 de	304.70 fg	456.73 ef
	EBL + Spm	1185.80 c–f	1295.40 cde	1498.13 bcd	278.80 efg	339.13 bcd	396.10 fg
	EBL + Si	1210.77 cde	1350.50 bcd	1505.17 bcd	327.27 bc	352.47 bc	485.70 de
	Spm + Si	1159.57 d–g	1214.93 efg	1562.73 bc	305.77 fgh	331.67 de	466.97 def
	EBL + Spm + Si	1331.03 ab	1385.13 bc	1606.10 b	341.17 ab	420.00 a	624.77 ab
Interrupted irrigation at the filling seed stage	Control	1126.20 efg	1154.83 fgh	1342.97 fgh	238.63 ij	280.30 h	380.80 fg
	EBL	1174.50 c–f	1194.27 efg	1472.63 cde	286.17 ef	333.70 cde	456.07 ef
	Spm	1170.67 c–f	1282.80 cde	1463.37 cde	289.03 ef	295.93 fgh	547.07 bcd
	Si	1237.03 cd	1219.77 efg	1445.57 def	284.97 ef	336.40 bcd	463.67 def
	EBL + Spm	1216.73 cde	1256.40 def	1501.87 bcd	259.40 ghi	328.90 de	463.67 def
	EBL + Si	1259.73 bc	1361.30 bcd	1535.80 bcd	312.07 cd	314.93 ef	533.70 cde
	Spm + Si	1351.40 ab	1432.70 b	1766.67 a	290.53 ef	331.30 de	604.47 abc
	EBL + Spm + Si	1409.23 a	1633.40 a	1842.77 a	348.93 a	400.27 a	667.50 a
LSD_5%_		92.46	121.51	110.96	21.24	20.51	86.37

Values shown in table are the means of three replicates. Different letters indicate significant differences among treatments according to LSD test when *p* ≤ 0.05. Where: *EBL* 24-epibrassinolide, *Spm* spermine, and *Si* silicon.

**Figure 4:**
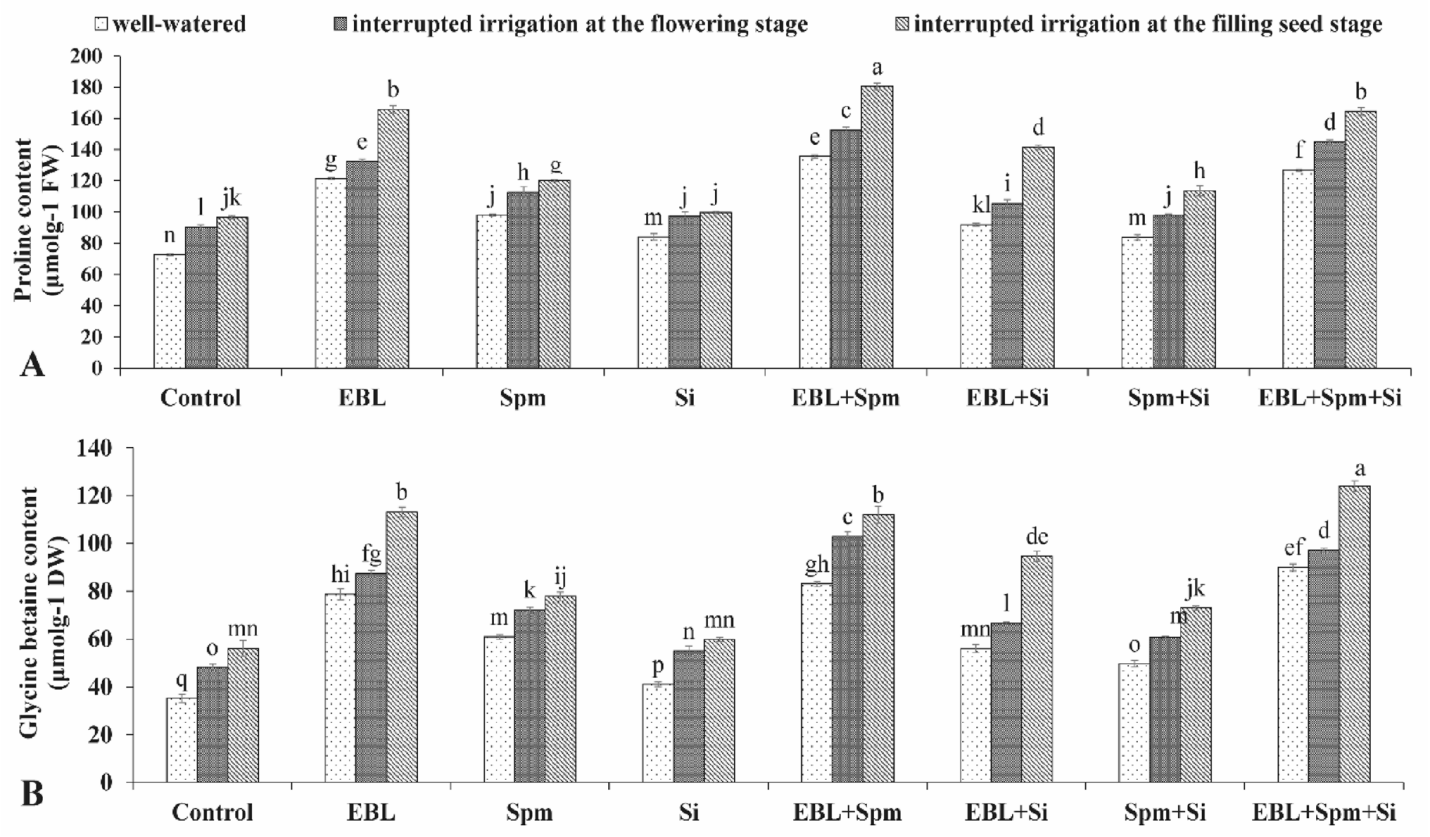
Changes in the proline (**A**) and glycine betaine contents (**B**) were exposed for various foliar applications [00.00 (double distilled water), 0.1 mg L^−1^ EBL, 25 mg L^−1^ Spm, 7 mg L^−1^ Si, 0.1 mg L^−1^ EBL + 25 mg L^−1^ Spm, 0.1 mg L^−1^ EBL + 7 mg L^−1^ Si, 25 mg L^−1^ Spm + 7 mg L^−1^ Si, and 0.1 mg L^−1^ EBL + 25 mg L^−1^ Spm + 7 mg L^−1^ Si] under different water deficit stress (well-watered condition and water-stressed conditions as interrupted irrigation at the flowering and the filling seed stages). Value shown in each column is the mean of three replicates ± standard error. Different letters indicate significant differences among treatments according to LSD test when p ≤ 0.05. Where: *EBL* 24-epibrassinolide, *Spm* spermine, and *Si* silicon.

Free proline and glycine betaine levels increased under drought circumstances, and this was more pronounced in stress-exposed plants treated with EBL, Spm, and/or Si (Fig. 4). Compared to untreated plants, the most effective treatment of [EBL (0.1 mg L^−1^) + Spm (25 mg L^−1^) + Si (7 mg L^−1^)] improved proline by 24.6%, 34.6%, and 51.3% and glycine betaine by 19.4%, 28.6%, and 49.3% in the leaves under non-stressed and stressed conditions of interrupted irrigation at the flowering and the filling seed stages, respectively.

MDA and H_2_O_2_ levels were increased (*p* < 0.05) under water stress (Table 2). However, the examination of these parameters in well-watered and water-deficient circumstances with the addition of EBL, Spm, and/or Si indicated a distinct response pattern. Under the non-stressed conditions, foliar application decreased MDA and H_2_O_2_ contents. Furthermore, treating the stressed plants with EBL, Spm, and/or Si led to a considerable decrease in the MDA and H_2_O_2_ contents. Under non-stressed and stressed compared to non-treated circumstances, respectively, triad treatment successfully reduced MDA content in maize leaves by 36.6% and 55.4% and H_2_O_2_ content by 38.6% and 58.2%, respectively.

**Table 2:** Changes in the of content malondialdehyde (MDA) and hydrogen peroxide (H_2_O_2_) were exposed for various foliar applications [00.00 (double distilled water), 0.1 mg L^−1^ EBL, 25 mg L^−1^ Spm, 7 mg L^−1^ Si, 0.1 mg L^−1^ EBL + 25 mg L^−1^ Spm, 0.1 mg L^−1^ EBL + 7 mg L^−1^ Si, 25 mg L^−1^ Spm + 7 mg L^−1^ Si, and 0.1 mg L^−1^ EBL + 25 mg L^−1^ Spm + 7 mg L^−1^ Si] under different water deficit stress (well-watered condition and water-stressed conditions as interrupted irrigation at the flowering and the filling seed stages).

Water stress	Foliar application	MDA (nmol TBARS g^−1^ FW)	H_2_O_2_ (nmol g^−1^ FW)
Well-watered	Control	192.43 ± 0.73 ef	292.47 ± 1.04 cd
	EBL	163.50 ± 1.53 l	248.57 ± 2.67 k
	Spm	173.13 ± 1.74 k	263.17 ± 3.10 ij
	Si	176.97 ± 0.81 jk	269.13 ± 2.30 hij
	EBL + Spm	135.03 ± 2.35 n	204.93 ± 1.57 mn
	EBL + Si	161.77 ± 0.87 l	245.80 ± 1.36 k
	Spm + Si	171.20 ± 1.42 k	260.23 ± 1.45 j
	EBL + Spm + Si	119.27 ± 0.49 o	181.50 ± 3.76 p
Interrupted irrigation at the flowering stage	Control	213.07 ± 1.39 b	330.13 ± 3.53 b
	EBL	181.33 ± 2.28 hi	280.40 ± 4.56 fg
	Spm	181.23 ± 2.93 hi	280.40 ± 3.71 fg
	Si	185.43 ± 1.91 gh	287.40 ± 1.40 def
	EBL + Spm	138.47 ± 1.04 n	214.70 ± 2.04 lm
	EBL + Si	176.00 ± 2.85 ijk	272.27 ± 2.95 ghi
	Spm + Si	178.93 ± 2.59 ij	277.27 ± 10.85 fgh
	EBL + Spm + Si	123.63 ± 1.02 o	191.43 ± 3.60 op
Interrupted irrigation at the filling seed stage	Control	241.27 ± 3.59 a	347.63 ± 3.51 a
	EBL	188.13 ± 3.54 fg	271.33 ± 6.03 ghi
	Spm	209.70 ± 1.25 b	302.43 ± 1.20 c
	Si	202.60 ± 0.80 c	291.97 ± 1.57 cde
	EBL + Spm	152.00 ± 3.43 m	218.93 ± 1.89 l
	EBL + Si	195.50 ± 0.67 de	281.40 ± 4.15 efg
	Spm + Si	198.00 ± 0.99 cd	285.07 ± 5.99 def
	EBL + Spm + Si	135.07 ± 2.49 n	194.90 ± 2.75 no
LSD_5%_		5.75	10.90

Values shown in table are the mean of three replicates ± standard error. Different letters indicate significant differences among treatments according to LSD test when *p* ≤ 0.05. Where: *EBL* 24-epibrassinolide, *Spm* spermine, and *Si* silicon.

## Discussion

Complex molecular and biochemical signal transduction pathways control plant responses to environmental stress and interact to determine tolerance or sensitivity at the whole plant level^20^. Plants respond to abiotic stress by altering the transcription, translation, and post-translational modification of defense-related genes and proteins, resulting in a sophisticated coordinated response to reprogram interconnected defense networks and metabolic processes^21^. Physiological and phenotypic responses have traditionally been the most important to capture in plant stress biology^22^. Modern research, on the other hand, involves identifying key genes that regulate stress tolerance and plant growth in a stressful environment, as well as screening gene function through knockout mutants or overexpression lines^20^.

The most typical sign of drought stress in plants is a decrease in the water content or potential of leaves. It stops the physiological functions of plants, such as photosynthesis, and results in plant death^23^. Several mechanisms control a plant's ability to survive a drought; among these, plant hormones always play a part^24^. Despite their biological functions, plant growth regulators play a crucial role under abiotic or biotic stressors^24^. A better path to understanding plant growth regulators under drought is provided by advancements in transcriptome and mutant analyses^25^. The two hormones, ABA and ethylene, have been extensively investigated to date and have shown activation and provision of tolerance under conditions of low water deficit^26,27^. During drought, the other key hormones, including auxin, CKs, and GA, also play a substantial role, albeit the molecular processes behind these hormones are only poorly known^28,29^. In addition, the other growth regulators such as brassinosteroids, SA, and jasmonic acid (JA), are also crucial in coping with drought^30^.

It disrupts the balance of antioxidant defenses and ROS quantity, leading to oxidative stress^31–33^. Against water stress, plants adopt numerous defense procedures of response or adaptation. The phytohormones brassinosteroids, polyamines, and silicon are evidenced to control the protective responses to drought in the maize plant^8,34,35^. Under different conditions 24-Epibrassinolide, spermine, and silicon regulate enzymatic and non-enzymatic antioxidants in the plant^8,36^. Improvement of the maize tolerance to drought through applying EBL, Spm, and/or Si is a great contribution. In this study, the negative effects caused by drought were lessened with the triad application of EBL, Spm, and Si. The current research will provide new information on how the use of the triad under water-stress settings might boost plant productivity. The grain yield and plant height were considerably (*p* < 0.05) impaired by the drought condition. However, the use of EBL, Spm, and/or Si greatly increased plant growth and output and mitigated the negative effects of the drought (Fig. 1). This result corroborated the findings of Desoky et al.^34^, Parveen et al.^8^, and Talaat^36^. Poor growth during droughts may be caused by excessive ROS production, which damages lipids oxidatively and raises the MDA concentration (Table 2). Excessive ROS generation results in loss of biomass due to multiple damages to the DNA, cellular membrane, pigments, proteins, lipids, and other vital components^37^. Against the adverse effects of drought, triad application of EBL, Spm, and/or Si enhanced plant growth and production. The improved plant development by the combinatory treatment under water stress is an external index of internal metabolic modifications. By applying foliar treatments, drought stress may be effectively induced. This is correlated with increased CAT, POX, and SOD activity (Fig. S1), high levels of total soluble protein and phenolic content (Fig. 3), low levels of H_2_O_2_ and MDA (Table 2), and better generation of organic solutes (Fig. 4). The information from this research suggests that the treated plants' detoxification processes included several metabolic pathways that included activating the antioxidant machinery.

Moreover, drought stress significantly reduces the relative water content (Fig. 2). However, foliar application of EBL, Spm, and/or Si ameliorated the RWC loss under drought stress. This was consistent with the finding of Anjum et al.^38^ and Talaat and Shawky^1^. The plants' ability to maintain their high RWC in water-scarce circumstances by using exogenous applications is connected to their participation in osmoregulation via an increase in proline and glycine betaine content (Fig. 4). Chlorophyll index is remarkably reduced (*p* < 0.05) by water stress (Fig. 2). Applications of EBL, Spm, and/or Si significantly reduced (p 0.05) the deleterious impacts of the drought by raising the chlorophyll index. It implies that the exogenous therapies might lessen the harm that drought causes to the chloroplast structure. This result corroborates the findings of Talaat^36^ and Parveen et al.^8^.

To face high ROS levels under stress conditions, plants develop a compensatory phenomenon as production of a wide range of non-enzymatic antioxidants (e.g. phenol derivatives)^39^. Normally, stressed plants create phenolic compounds; however, when treated with EBL, Spm, and/or Si, they accumulate even more phenols, showing their limited antioxidant activity in the plant. In this regard, Desoky et al.^34^ and Talaat and Shawky^1^ highlighted that the improvement of tolerance to stress conditions by EBL, Spm, and Si supplementation is due to the role of phenolic compounds.

An oxidative explosion is caused by a buildup of ROS brought on by drought conditions. The balance between ROS generation and its scavenging is maintained by a variety of antioxidant enzymes. Increased activity of antioxidant enzymes is established to promote plant protection against stress Ashraf^37^. Under drought stress, there was evidence of increased SOD, POX, and CAT isoform activity, which is consistent with the finding of Noein and Soleymani^31^. However, the increased enzyme activity did not provide enough defence against damaging ROS and the consequent oxidative damage, as shown by the contemporaneous increase in MDA and H_2_O_2_ levels (Table 2). Application of EBL, Spm, and/or Si in the stressed plants seems to develop a reservoir of the antioxidant enzymes (Table 1 and Fig. 3). Improved activities of CAT, POX, and SOD effectively scavenge ROS; this was supported by a remarkable decrease of H_2_O_2_ and MDA levels in the maize (Table 2). Similar observation has been reported by Anjum et al.^38^ and Parveen et al.^8^. Under drought stress, a considerable increase occurs in MDA level as a marker of membrane lipid peroxidation^37^ which has an appropriate correlation with H_2_O_2_ production (Table 1). Decreased MDA and H_2_O_2_ accumulation were measured in maize leaves under the water stressed condition. The lower level of MDA and H_2_O_2_ could be due to increased stress tolerance in the maize plant. This outcome demonstrated that the treated plants were more resilient to oxidative stress and less sensitive to drought. Treatments result in a much decreased MDA level in stressed plants compared to untreated circumstances. This might be indicative of the lower electrolyte leakage in the treated in comparison to untreated plants, which is confirmed by the literature^4,8,38^.

Compatible solutes, in addition to antioxidant enzymes, actively attenuate the adverse effects of drought stress. Proline and glycine betaine concentration increased in response to a water-stress condition (Fig. 4), which might be interpreted as an attempt to regulate osmotic pressure in order to reduce water loss in the plant. Further, the application of EBL, Spm, and/or Si significantly stimulated the overproduction of the organic solutes in the stressed plants. This data was in accordance with the findings of Anjum et al.^38^, Talaat et al.^4^, Talaat and Shawky^1^, and Parveen et al.^8^. In stressed plants, the enhanced accumulation of proline and glycine betaine protects membrane integrity, neutralises free radicals, reduces membrane lipid oxidation, keeps enzymes that neutralise ROS active, stabilises subcellular structures, and keeps the redox balance stable^37,40^. Considering the interaction between the three treatments, exogenous application of EBL and/or Spm was shown to cooperate effectively with exogenous Si in stressed plants inducing their growth as well as productivity, the antioxidant activity of the enzymes, and production of osmoprotectants. The significant increment of the growth and measured parameters by the combinatory treatments of the phytohormones was higher than that of the single treatments.

Our work brings a new eco-physiological aspect to the field since it is based on a complex of features measurements (biochemical, physiological, and growth) that are applied together to understand the EBL, Spm, and Si effect in plant response to stress conditions. Such an approach states a general testing protocol and allows a better understanding of plant response to changes in water quantity, nevertheless of the tested species.

## Materials and methods

### Experiment design, growth condition, and water stress

Hybrid maize, namely cultivar cv. Paya was prepared by Ardabil Agricultural and Natural Resources Research and Education Center (Moghan, Iran). This genotype was chosen for this study based on its improved productivity. Our studies are complied with the relevant institutional, national, and international guidelines and legislation. The plant experiments complied with local and national regulations and following Ardabil Agricultural and Natural Resources Research and Education Center (Moghan, Iran) regulations. All experiments were conducted in Agricultural Research Station of Moghan (Moghan, Iran). We have permission to collect the Hybrid maize seeds from Ardabil Agricultural and Natural Resources Research and Education Center (Moghan, Iran).

A field trial was performed during 2020–2021 at the Agricultural Research Station of Moghan. Randomized complete block design with two factors was considered in the experiment with three irrigation regimes of well-watered, water-stressed, and interrupted irrigated conditions at the flowering and the filling seed stages. Further, at all irrigation regimes, the plants were exposed to eight spraying treatments of double distilled water, 24-epibrassinolide (EBL) (0.1 mg L^−1^), spermine (Spm) (25 mg L^−1^), and silicon (Si) (7 mg L^−1^), 24-epibrassinolide (EBL) (0.1 mg L^−1^) + spermine (Spm) (25 mg L^−1^), 24-epibrassinolide (EBL) (0.1 mg L^−1^) + silicon (Si) (7 mg L^−1^), spermine (Spm) (25 mg L^−1^) + silicon (Si) (7 mg L^−1^), and 24-epibrassinolide (EBL) (0.1 mg L^−1^) + spermine (Spm) (25 mg L^−1^) + silicon (Si) (7 mg L^−1^). In total, twenty-four treatments, each with three replicates were considered in this experiment. The experiment plots contained four rows (3 m × 0.75 m) with the soil type of sandy-loam composed of 49.8% sand and 18.5% clay. At the planting stage, nitrogen fertilizer was applied as 60 kg ha^−1^. Four weeks after cultivation, 60 kg ha^−1^ nitrogen was excessively added.

EBL and Spm (both Sigma, USA) were respectively dissolved in an appropriate volume of ethanol and distilled water. Moreover, Si (Na_2_SiO_3_, MW = 122.1) was purchased from Arya Chemical Co. (Iran) and was dissolved in distilled water. An initial screening was carried out for different dilutions of EBL, Spm, and Si to get the ideal plant responses. Based on this, the concentration levels of 0.1 mg L^−1^ EBL, 25 mg L^−1^ Spm, and 7 mg L^−1^ Si were chosen. Foliar spraying of EBL, Spm, and Si, was performed at the flowering and filling seed stages of the maize plants. 20 days following the application of the mentioned treatments, plant samples were collected.

### Determination of the content of hydrogen peroxide (H_2_O_2_)

The H_2_O_2_ content was measured according to the methods presented by Talaat et al.^4^. To homogenize the leaf tissues, a phosphate buffer of 50 mM (pH adjusted to 6.8) was used (3.0 mL phosphate buffer per 0.5 g leaf). Centrifugation of the homogenate was performed for 25 min at 6000×*g*. Titanium chloride in sulphuric acid (20% v/v) was added to the extract as 0.1% and the mixture was centrifuged for 15 min at 6000×*g*. The H_2_O_2_ content was measured spectrophotometrically at 410 nm. For each duplicate, three biological replications from the newest fully grown leaves were obtained from three distinct plants. Based on the absorbance values against the known H_2_O_2_ concentrations, the standard curve was established.

### Determination of lipid peroxidation

Equivalents of malondialdehyde (MDA) content were expressed as presentative of the oxidative damage to lipids. Homogenization of fresh leaves (0.5 g) was performed in ethanol (80%). Then centrifugation was performed at 4 °C, for 10 min at 3000×*g*. Following two-fold extraction of the pellet with ethanol (80%), it was transferred to a new test tube. The mixture solution of butylated hydroxy toluene (0.01%), thiobarbituric acid (0.65%) and trichloroacetic acid (20%) was prepared and added in an equal volume to the supernatant from the previous step. The mixture was incubated at 95 °C for 25 min and afterwards it was cooled down to room temperature. The spectrophotometric measurements were made at the wavelengths of 440, 532, and 600 nm, and the values were used in MDA calculation formula^41^. For each duplicate, three biological replications from the newest fully grown leaves were obtained from three distinct plants.

### Electrophoresis, protein content, and antioxidant enzymes

For each duplicate, three biological replications from the newest fully grown leaves were obtained from three distinct plants. Preparation of crude leaf extracts was performed in Tris–HCl extraction buffer^42^ with the ratio of 1 W:2 V and centrifuged at 4 °C for 10 min at 10,000*g*. A filter paper was applied to directly absorb enzyme extracts. The extracts were electrophoresed on horizontal slab polyacrylamide gel (7.5%) using electrode buffer of Tris–Borate-EDTA (TBE; pH = 8.8) for 4 h at 4 °C. Following electrophoresis, two slices were excised from the slab gels and were stained to evaluate the enzyme activities. To determine superoxide dismutase (SOD), peroxidase (POX), as well as catalase (CAT) staining, was performed respectively according to the protocol by Moharramnejad and Valizadeh^42^ and Moharramnejad et al.^2^. The stained gels were then fixed and scanned at an appropriate range of wavelengths. To calculate optical density × area for each isozyme activity the image analysis software, MCID, was used. The total protein content related to the enzyme extracts was measured following Bradford^43^.

### Determination of total phenolics

Three biological replications from the most recent fully developed leaves of three different plants were acquired for each duplicate. Homogenization of 50 mg of fresh leaf tissue was carried out with acetone (80%) and then centrifugation was performed for 10 min at 10,000*g*. Water (2 mL) and Folin–Ciocalteau’s phenol reagent (1 mL) was added to dilute 100 µL of the supernatant and the mixture was vigorously shaken. Addition of sodium carbonate solution (20%) was performed as 5 mL and then distilled water was used to reach the whole volume to 10 mL. Following a thorough mixing, the absorbance was measured at the wavelength of 750 nm^40^. The calculations were made to report the final data as mg/g of fresh leaf.

### Determination of glycine betaine and proline contents

From the newest fully grown leaves were taken glycine betaine was extracted with 3 biological replicates for each duplicate as described by Grieve and Grattan^44^. The mixture of toluene–water (0.5% toluene) was used to treat dried ground leaves (as 5 mL per 0.1 g). After 24 h shaking at 25 °C, the extracts were filtered and the volume was made up to 100 mL. To 1 mL of the solution, 2 N HCl (1 mL) was added. Potassium triiodide solution (0.1 mL) was used to treat 0.5 mL of the solution taken from the extract in the previous step. After 90 min shaking in the ice bath, ice-cooled water (2 mL) and 1,2-dichloroethane (4 mL) was added. When stirred two phases were separated in the mixture; the lower phase was isolated and the OD at 365 nm was measured. To determine proline content the method of Bates et al.^45^ was used. The extract of the fresh leaf was prepared by sulphosalicylic acid, ninhydrin glacial, and acetic acid solutions. After incubating the samples at 100 °C, toluene (5 mL) was added. Toluene phase was transferred to a new container and the OD was measured at 528 nm. A standard curve was established based on the absorbance of different concentrations of proline (Sigma, USA).

### Determination of relative water content (RWC)

Determination of RWC was performed using 2 cm^2^ diameter disks of fresh leaf. After weighing to saturate the leaf disks, they were floated on double-distilled water and maintained in dark for the next 24 h. Followed by blotting the disk adhering water, turgor mass was determined. Dehydration was carried out at 70 °C for 48 h, then disk dry mass was measured. Calculation of RWC was performed based on the formula below:

$$ {\text{RWC}} = \left( {{\text{Fresh mass }} - {\text{dry mass}}{/}{\text{Turgor mass }} - {\text{dry mass}}} \right) \times 100. $$

### Chlorophyll index

The chlorophyll index of the fresh leaves was determined by SPAD-502 Chlorophyll Meter (Ramsey, USA). The chlorophyll meter's performance was calibrated in accordance with the manufacturer's instructions prior to obtaining the measurements. Six readings from each replication were collected at the measurement date using the newest fully grown leaves.

### Statistical analysis

Randomized complete block design with the two-factor factorial arrangement and three replicates were considered for the trial experiment. The ANOVA and significant differences among the mean values were analyzed by the Least-Significant-Difference (LSD) with a confidence interval of 95%.

### Statement permission

The authors declare that they have proper permission.

## Conclusions

The effectiveness of the trio of EBL, Spm, and Si in the plant response to a stress state was disclosed in the current research, which provides important light on the function of phytohormones while restricting their use in agriculture. Maize tolerance to drought was enhanced by dual/triad application through the induced high antioxidant activity of the enzymes to buffer cellular redox potential, leading to limited H_2_O_2_ accumulation and restricted lipid peroxidation. Therefore, dual/triad application is a potential approach to enhance plant production in areas with drought soil. Dual/triad application may also function well as an immuno-modulator if used at the right dose and stage of plant growth. The current research points us in the direction of the unknown mechanisms by which the dual/triad treatment increases plant drought resistance.

## Supplementary Information

Supplementary Figure S1.

## Data Availability

All data available within the article and supplementary information file.
