# Supplementary material for: Impact of 24-epibrassinolide, spermine, and silicon on plant growth, antioxidant defense systems, and osmolyte accumulation of maize under water stress
Source: Sci Rep. 2022 Aug 27;12:14648. doi: 10.1038/s41598-022-18229-1 (PMC9420132; doi:10.1038/s41598-022-18229-1)
Supplement: Supplementary file 1 — Supplementary Figure S1. [file 41598_2022_18229_MOESM1_ESM.pdf]

Supplementary data

Original research manuscript submitted to Scientific reports

Impact of 24-epibrassinolide, spermine, and silicon on plant growth, antioxidant defense systems, and osmolyte accumulation of maize under water stress

Azizolah Ghasemi [1], Salim Farzaneh [1,*], Sajjad Moharramnejad [2,*], Raouf Seyed Sharifi [1], , Ahmed F. Youesf [3,*], Arkadiusz Telesinski [4], Hazem M. Kalaji [5,6], Jacek Mojski[7,8]

[1] Department of Genetics and Plant Production, Faculty of Agriculture and Natural Resources, University of Mohaghegh Ardabili, Ardabil, Iran

[2] Crop and Horticultural Science Research Department, Ardabil Agricultural and Natural Resources Research and Education Center, AREEO, Moghan, Iran

[3] Department of Horticulture, College of Agriculture, University of Al-Azhar (Branch Assiut), Assiut 71524, Egypt

[4] Department of Bioengineering, West Pomeranian University of Technology in Szczecin, 17 Słowackiego Street, 71-434 Szczecin, Poland.

[5] Department of Plant Physiology, Institute of Biology, Warsaw University of Life Sciences SGGW, 02-776 Warsaw, Poland

[6] Institute of Technology and Life Sciences, National Research Institute, Falenty, Al. Hrabska 3, 05-090 Raszyn, Poland

[7] Twój Swiat Jacek Mojski, ulica Okrzei 39, 21-400 Lukow, Poland

[8] Fundacja Zielona Infrastruktura, ulica Wiatraki 3E, 21-400 Lukow, Poland

***** Correspondence: salimfarzaneh@yahoo.com (S.F.); sm.chakherlo@yahoo.com (S.M.)

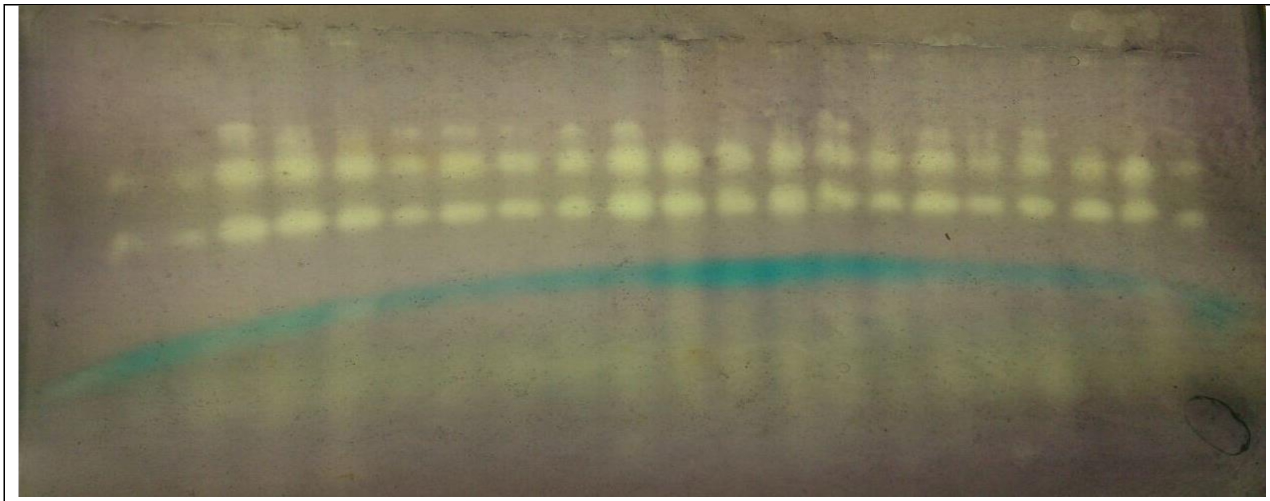

SOD3

SOD2

SOD1

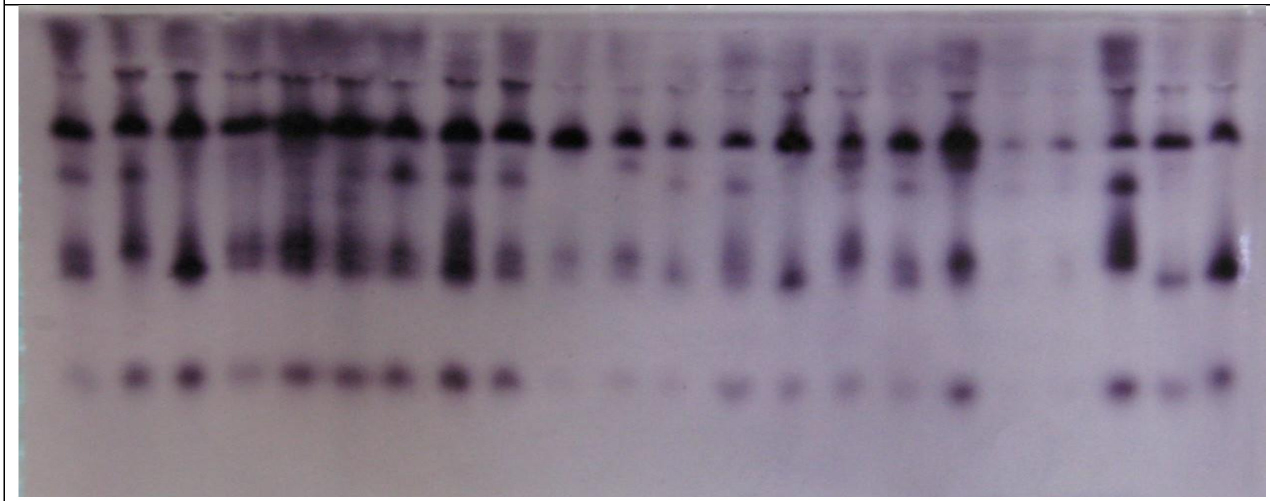

POX2

POX1

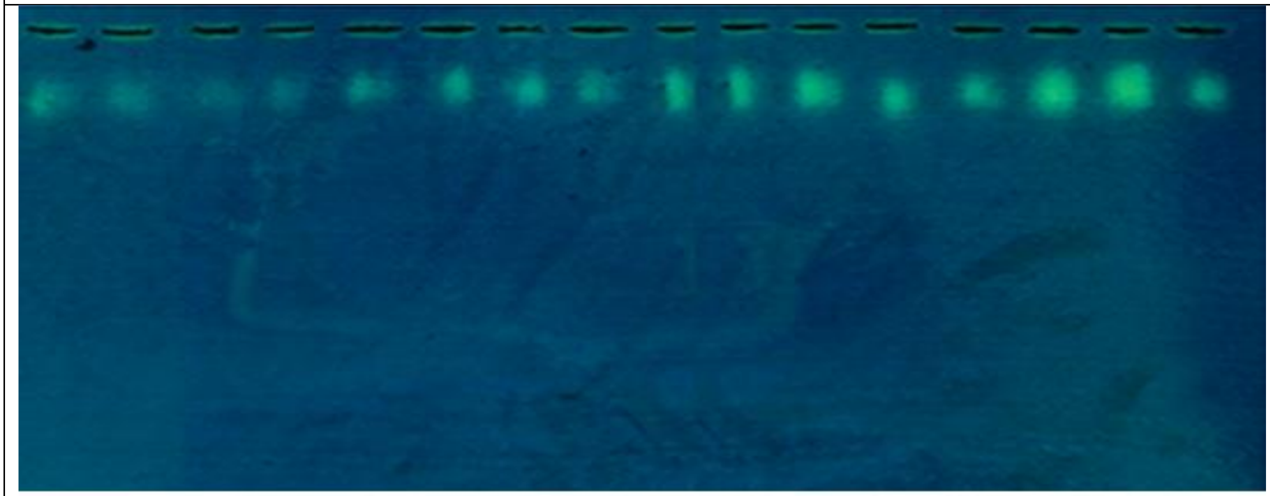

CAT

Figure S1. The banding pattern of superoxide dismutase (SOD), peroxidase (POX), and catalase (CAT) isoforms onto polyacrylamide gels in the maize.
